# Nexobrid Use in the Elderly

**DOI:** 10.3390/ebj6040058

**Published:** 2025-11-07

**Authors:** Alexander Lugilde Guerbek, Jordi Serracanta Domenech, Antonio Bulla, José Antonio López Martínez, Danilo Rivas Nicolls, Alex Arteaga, Alejandro Grabosky Elbaile, Sara Orois, J. P. Barret

**Affiliations:** Vall d’Hebron Burns Unit, Plastic Surgery Department, 08035 Barcelona, Spain; jordi.serracanta@vallhebron.cat (J.S.D.); alejandro.grabosky@vallhebron.cat (A.G.E.); juanpedro.barret@vallhebron.cat (J.P.B.)

**Keywords:** Nexobrid, elderly, experience

## Abstract

Background: Enzymatic debridement with Nexobrid (NXB) is established for burn care, but specific outcomes in the elderly remain poorly characterized. This study evaluates the safety, efficacy, and clinical outcomes of NXB in patients aged ≥65 years. Methods: A retrospective case-series of 43 consecutive elderly patients (mean age 74.5 years) with deep partial- to full-thickness burns treated with NXB at a single burn center. Data on demographics, burn characteristics, treatment chronology, and complications were analyzed. Results: The median total burn surface area (TBSA) was 11%. NXB was applied selectively, with a mean debrided area of 7.41% TBSA, primarily on limbs and hands. While 76.7% of patients ultimately required surgical autografting, no patient required an escharotomy in NXB-treated areas. The mortality rate was 25.6%, which was lower than expected for a median revised Baux score of 90, which is expected to be more than 50%. Hypertrophic scarring occurred in 28.1% of survivors, associated with a prolonged median healing time of 63 days. Conclusions: In elderly burn patients, NXB facilitates precise eschar removal and reliably prevents compartment syndrome, demonstrating a strong safety profile even in high-risk individuals. Its primary benefit shifts from reducing surgical incidence to optimizing the wound bed for grafting. These findings support the use of NXB in the elderly, with the understanding that subsequent grafting is often still required due to age-related delayed healing.

## 1. Introduction

Nexobrid^®^ (NXB) is an enzymatic debriding agent derived from *Ananas comosus* (pineapple) that contains a mixture of proteolytic enzymes, primarily bromelain. It is manufactured by MediWound Ltd. (Yavne, Israel) and distributed in Europe by Vericel Corporation. NXB is approved for the removal of eschar in deep-partial and full-thickness thermal burns in patients of all ages [1]. The product acts by selectively dissolving non-viable burn tissue while preserving viable dermis, thereby allowing a more accurate assessment of burn depth and potentially facilitating spontaneous healing.

Studies have demonstrated that NXB can significantly reduce the need for surgical excision and blood transfusion compared to conventional tangential excision [2]. The body of literature on NXB is growing, with studies increasingly focusing on its use in specific patient subgroups. For instance, a recent open-label randomized controlled trial by Shoham et al. (2025) confirmed the efficacy and safety of NXB in the pediatric population, demonstrating its superiority over standard of care by significantly reducing the time to complete eschar removal (1 vs. 6 days), and drastically minimizing the need for surgical excision (1.5% vs. 48.1% of wound area) [3]. Similarly, a recent multi-center study by Tapking et al. (2023) has begun to address the gap in geriatric burn care, confirming that enzymatic debridement is a reliable and safe method for selective eschar removal in geriatric patients, with no significant effect of age on the need for subsequent surgery [4].

However, the European Medicines Agency (EMA) notes that clinical experience in elderly patients (>65 years) is limited, and treatment in this population requires careful assessment of its risks and benefits [5]. Elderly burn patients represent a particularly fragile group, with decreased physiological reserve, higher rates of comorbidities, and increased susceptibility to complications such as infection, delayed wound healing, and mortality. Despite advances in burn care, outcomes in this demographic remain significantly worse than in younger populations. The optimization of non-surgical, tissue-sparing debridement methods like NXB may hold special value for elderly patients by minimizing operative stress and transfusion-related risks [6,7,8].

Our Burns Center at Vall d’Hebron Hospital Campus has extensive experience using NXB across a wide range of ages and burn severities, having participated in international consensus guidelines regarding its clinical use [9]. A prior institutional study involving 300 adult patients (median age 41 years) confirmed that enzymatic debridement (ED) is a reliable, rapid, and tissue-preserving method for eschar removal, aiding in accurate depth evaluation and helping avoid unnecessary surgeries, especially in functional areas such as the hands and upper limbs [10]. However, the specific outcomes and safety profile of NXB in elderly patients have not been independently analyzed.

While the study by Tapking et al. established the efficacy of the debridement phase, evidence regarding the subsequent recovery and long-term outcomes in the elderly remains sparse. Given the need for more detailed data on the clinical course post-debridement, this study aims to provide a granular evaluation of NXB’s use in this fragile population, focusing not only on initial efficacy and safety but also on long-term healing, hospitalization metrics, and the outcomes of surgical grafting. We hypothesize that NXB will facilitate a recovery characterized by reduced transfusion requirements and infection rates. However, higher mortality is expected compared to younger cohorts, likely reflecting age-related frailty and burn severity rather than NXB itself.

The findings from this study may provide evidence-based insights into NXB use in elderly burn patients, enhancing advanced training and clinical decision-making. Furthermore, it lays the groundwork for future prospective studies to support NXB’s safety and effectiveness in this specific demographic.

## 2. Materials and Methods

The study was carried out taking into account the principles of the Declaration of Helsinki and with the approval of the Vall d’Hebron Ethics Committee (Protocol number: EOM(AG)027/2023(6136)). All patients signed their consent on admission for the procedure and the collection of their data.

All patients were treated at “Vall d’Hebron Hospital” Burns Center (Barcelona). At the time of admission, a plastic surgeon evaluated the percentage of Total burned body surface area (TBSA) and assessed burn depth. Clinical assessment was based on established visual and tactile characteristics. For this study, NXB was indicated and applied to areas presenting with intermediate/deep partial-thickness (deep second-degree) or full-thickness (third-degree) burns. Deep partial-thickness burns were identified by their pale, mottled, or cherry red appearance, with diminished capillary refill and sensation. Full-thickness burns presented as dry, leathery and insensate, often with a waxy white or charred appearance. It is important to note that burn wounds frequently exhibit mixed depth, and the clinical assessment targeted the deep and intermediate areas for enzymatic debridement. A critical care doctor assessed if further intensive management was needed. All patients underwent a standardized diagnostic protocol, which included chest radiography, a comprehensive laboratory panel (complete blood count, coagulation profile, liver and renal function tests, serum albumin, calcium, and lactate), and serological screening for hepatitis B, hepatitis C and HIV. In patients requiring fluid resuscitation metabolic status was monitored with serial venous blood gas analyses. The patients received fluid resuscitation according to the Parkland formula.

The decision to administer blood products (red blood cells (RBC), fresh frozen plasma (FFP), platelets) was based on a comprehensive clinical assessment rather than a single laboratory value. Guidelines for transfusion included a hemoglobin level persistently below 7–8 g/dL, particularly in the context of active bleeding, anticipated major surgical debridement, or clinical signs of impaired oxygen delivery (e.g., unexplained tachycardia or hypotension). Coagulation products were administered for documented coagulopathy (INR > 1.5) or thrombocytopenia (platelet count < 50,000/μL) associated with active bleeding or prior to an invasive procedure.

During the initial wound cleansing any loose, necrotic epidermal tissue and blisters were gently removed. The wound was then covered with temporary wet gauze dressings saturated with Prontosan^®^ (a polyhexanide-based irrigation solution) and Microdacyn^®^ (a hypochlorous acid-based spray) until the NXB therapy was initiated.

NXB was applied up to 4 days after burn suffering. In the meantime the same wet dressing protocol was used. Analgesia and sedation were managed following a standardized protocol. The procedure was performed under either sedation (using Propofol, Midazolam, or Ketamine) with regional nerve blocks (Ropivacaine) for limb burns, or general anesthesia for facial burns. After layering NXB in an occlusive manner and applying vaseline on the edges to avoid spilling, it was left to debride for 4 h and then removed in a sterile manner. Finally, a post-NXB burns depth evaluation was carried out up to 24 h after removal, with the application of a follow-up dressing over the area. A multimodal analgesic regimen was maintained throughout the hospital stay. It included scheduled Paracetamol and NSAIDs for background pain, with supplemental opioids (e.g., Morphine or Fentanyl) for breakthrough pain during procedures or dressing changes.

Following enzymatic debridement with NXB, the wound bed was subsequently prepared for grafting using hydro-debridement to thoroughly clean the area and remove any residual debris or non-viable tissue. This step was carefully performed to ensure a clean, viable graft bed while avoiding over-debridement. Definitive closure of deep burns in elderly patients was then achieved using split-thickness autografts. Autografts were harvested at a thickness of 0.010 to 0.015 inches from available donor sites using a pneumatic dermatome. Depending on the specific wound characteristics and patient factors, the autografts were applied either meshed (1.5:1 ratio) or laminar (unmeshed). The grafts were secured in place with surgical staples or sutures and covered with a non-adherent, antimicrobial dressing. Donor sites were managed with a standard protocol of a semi-occlusive, absorbent dressing to facilitate re-epithelialization.

We collected data from clinical history about age, sex, etiology, TBSA, previous comorbidities, inhalation injury, need for intubation, surface area treated with NXB, time until NXB application, time from NXB application to surgery post-NXB dressing used, hospital stay length, mortality, revised Baux score, cause of death, infection, time-to-healing, need for surgery, escharotomy of NXB treated surfaces, need for transfusion (platelets, FFP, RBC used up until the first surgery), scar hypertrophy, pressure therapy use.

### Statistical Analysis

The results are summarized in a descriptive manner, applying relative frequencies for categorical variables, whereas means and medians were used for numerical ones.

A *post hoc* exploratory statistical analysis was performed to assess potential associations between mortality and patient comorbidities, coagulopathy-related variables, and other clinical parameters. Categorical variables included the presence of comorbidities (cardiac disease, diabetes mellitus, severe respiratory disease, malignancy, or neurologic disease), inhalation injury, intubation on admission, local infection, and transfusion requirement. Continuous variables included age, percentage of total body surface area burned (%TBSA), percentage of area treated with Nexobrid (%TBSA-NXB), number of transfused blood units, time from burn to NXB application, time from NXB to surgery, and time-to-healing. Given the limited sample size and the non-parametric distribution of the data, categorical variables were compared between survivors and deceased patients using Fisher’s exact test, and continuous variables using the Mann–Whitney U test.

## 3. Results

From January 2015 to August 2023, our Burn Unit admitted 255 patients aged 65 years or older. From this population, forty-three consecutive patients treated with NXB from November 2015 to August 2023 were included on a case-series basis. Their mean age was 74.48 years (Standard Deviation (SD) = 7.56, Range 66–94). There were thirteen women and thirty men (69.8%). The most frequent burn etiology was flame (83%), followed by scalding (14%) and contact (3%) burns. Four patients (9.3%) suffered inhalation injuries, while seven (16.3%) were intubated either at the emergency department or during transfer. The median burned TBSA was 11% (range 0.75–78%). The chronology of NXB treatment and subsequent wound management was as follows: the mean time from burn injury to NXB application was 1.16 days (SD = 1.13, Range 0–4). For the 33 patients (77%) who required surgical autografting following enzymatic debridement the mean time from NXB application to surgery was 13.77 days. The mean time from the initial burn injury to complete healing was 70.35 days. The mean percentage of NXB TBSA debrided was 7.41% (SD = 6.17, Range 0.75–32). Patient comorbidities are summarized in Table 1.

The median TBSA was significantly higher in non-survivors (34%) than in survivors (7%). The median percentage of TBSA treated with NXB was 8% in non-survivors versus 5.5% in survivors. The median revised Baux score was 90 overall, 109 in non-survivors, and 84 in survivors.

**Figure 1:**
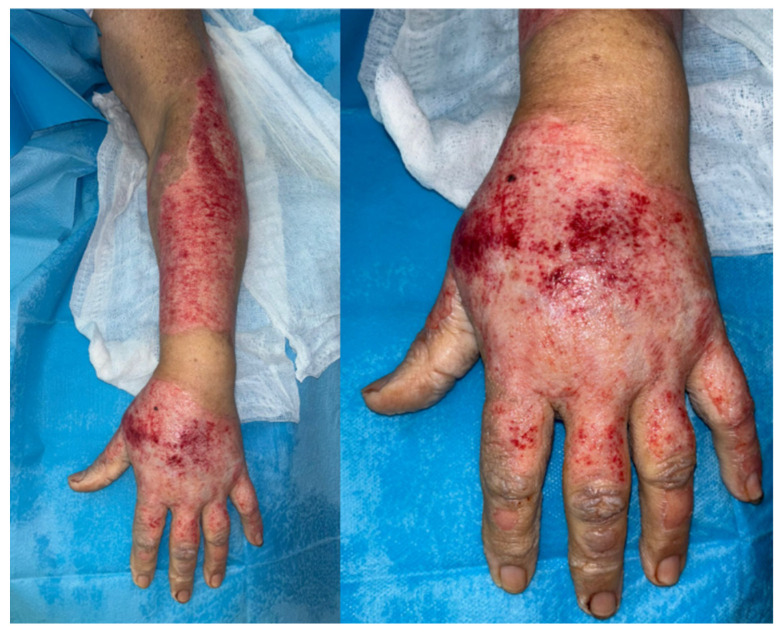
NXB debrided hand and forearm on a 71 year-old female, showing areas with deeper dermis loss on the dorsum of the hand and the “step sign” [10].

The most frequently debrided areas were the hands (n = 18), full upper limbs (right: n = 9; left: n = 7), and full lower limbs (right: n = 10; left: n = 12). Other areas included the face, forearm, arm, thorax, thigh, leg, and foot (Figure 1).

The most common post-enzymatic debridement dressing was a polyurethane-based wound contact layer with polylactic acid (Urgotul™ with Suprathel™) (n = 22), followed by a silicone mesh with nitrofurazone (Mepitel™ with Furacin™) (n = 13), silver sulfadiazine (n = 6), and a silver-impregnated soft silicone foam (Mepilex AG™) (n = 3). In one instance, a direct split-thickness autograft meshed at 1:1.5, of approximately 1% of TBSA, was applied. Other dressings were used in fewer cases.

Eleven patients (25.6%) received a transfusion: ten patients received RBC concentrates (median 3 units), four received fresh frozen plasma (median 2.5 units), and three received platelet transfusions (median 1 unit).

Eleven patients (25.6%) developed an infection affecting the NXB-treated area. The most frequently isolated microorganisms were *Pseudomonas* spp. (n = 6) and *Staphylococcus aureus* (n = 3). Other isolates are listed in Table 2.

Thirty-three patients (77%) required surgical coverage after NXB. No patient required an escharotomy in NXB-treated areas. The median hospital stay was 26.5 days (range 2–101). Among survivors twelve required pressure therapy and nine developed hypertrophic scarring in NXB-debrided areas.

Eleven patients (26%) died during their hospital stay. In univariate analyses larger %TBSA (median 34% in deceased vs. 7% in survivors; *p* < 0.001) and the need for transfusion (6/11 deceased vs. 5/32 survivors; *p* = 0.010) were significantly associated with mortality. No significant associations were found for the presence of specific comorbidities, inhalation injury, intubation on admission, local infection, or time-related variables. Causes of death are summarized in Table 3.

## 4. Discussion

This study provides a detailed analysis of enzymatic debridement with NXB in elderly burn patients, a population for whom specific outcome data remain limited despite EMA approval for all age groups [5]. Our findings suggest that while NXB may not reduce the ultimate need for surgical grafting in this demographic, it offers distinct advantages in safety and wound bed preparation that may optimize final outcomes.

Consistent with general burn epidemiology in younger adults, our cohort was predominantly male (69.8%) with flame as the primary etiology [11]. However, the median TBSA of 11% indicates NXB was typically used in non-severely burned patients. A key finding was the selective application of NXB; the mean debrided area (7.41% TBSA) was substantially less than the total burn size, reflecting a targeted approach to high-priority areas like the hands and limbs. The strategic application of enzymatic debridement is consistent with a treatment philosophy that leverages its capacity for enhanced wound bed assessment. This approach permits zones initially deemed to require surgical intervention an opportunity for maximal spontaneous healing, thereby minimizing the total body surface area (TBSA) subjected to surgical debridement and autografting. The selective preservation of viable tissue subsequently facilitates a more precise and potentially less hemorrhagic autografting procedure. This consideration is particularly crucial for optimizing functional recovery in elderly patients.

The clinical timeline in our cohort reflects a deliberate patient-centered approach. The mean time-to-NXB of 1.16 days is consistent with recommendations [1] and practice in other centers [4], confirming the feasibility of early application even in fragile patients. The subsequent mean interval of 13.77 days from NXB to surgery was carefully timed to maximize spontaneous healing and physiological optimization while avoiding prolonged delay and excessive scarring, before undertaking the stress of autografting.

The median hospital stay was 26.5 days, with a wide range (2–101 days) reflecting the spectrum of burn severity and corresponding care needs. A *post hoc* analysis clarified this distribution, revealing three distinct groups: non-grafted patients (including those with fatal burns) had a short median stay of 7.5 days; grafted patients without major complications had a median stay of 27.5 days; and grafted patients with significant complications had the longest median stay of 35 days. This stratification confirms that the overall median of 26.5 days robustly represents the typical hospitalization for an elderly burn patient who requires and survives surgical intervention.

This extended healing pathway is further illustrated by two key outcomes. First, despite optimal debridement 76.7% of patients required surgical grafting, a rate higher than the 58.8% reported by Tapking et al. [4]. We attribute this primarily to the inherently slower healing in elderly patients, which often precludes spontaneous healing of deep dermal injuries. Secondly, the mean total healing time of 70.35 days likely contributed to the development of hypertrophic scarring in 28.1% of survivors, a notable finding given that hypertrophic scarring is considered less common in the elderly [11]. This underscores that prolonged inflammation and re-epithelialization can predispose even older patients to abnormal scarring.

The safety of NXB in this fragile cohort is a critical finding. Most notably no patient required an escharotomy in NXB-treated limbs, demonstrating its efficacy in preventing compartment syndrome—a significant clinical advantage. Furthermore, the need for proactive airway management in severely injured patients can be leveraged to facilitate NXB application in complex areas. In one instance, a patient who arrived intubated for unrelated reasons underwent successful NXB debridement of deep facial burns within 24 h. This scenario highlights that pre-existing intubation, while indicating severity, can provide a unique opportunity to safely debride anatomically challenging areas like the face without requiring additional anesthetic procedures, optimizing the timing and quality of eschar removal.

The transfusion rate of 25.6% is likely multifactorial, attributable to the patients’ baseline anemia [12,13], their clinical condition, and the burden of large TBSA burns rather than the debridement method itself. Reassuringly and in line with recent evidence [14,15], we found no significant coagulopathy attributable to NXB, with minimal requirements for platelets or FFP.

The infection profile was as expected for a burn unit, with *Pseudomonas* spp. and *S. aureus* being most prevalent [16]. The appearance of opportunistic organisms like *Mucor* and *Candida* in a minority of cases highlights the immunocompromised state of some elderly burned patients.

The observed mortality rate of 25.6% must be interpreted within the context of burn severity. Non-survivors had a median TBSA of 34%, nearly five times that of survivors (7%). This is reflected in the revised Baux score, where the median score for the entire cohort was 90 (associated with a >50% mortality risk [17,18]), and non-survivors had a median score of 109 (typically associated with >90% mortality [17,18]). The fact that our observed mortality was lower than predicted by these high scores suggests that NXB can be safely applied to elderly patients with grave prognoses. This aligns with findings by Tapking et al., who also identified burn size and age and not the debridement method as the primary mortality drivers [4].

A key limitation of this study is its single-arm, descriptive design. As NXB is the standard of care for eligible patients in our unit, constructing a comparable internal control group is methodologically unsound. Therefore, to quantitatively understand the effect of NXB on survival, blood loss, and functional outcomes, future prospective, multi-center studies with carefully matched control groups are necessary.

## 5. Conclusions

NXB represents a valuable and safe debridement tool in the care of elderly burn patients. Its primary benefit in this population may shift from reducing surgical incidence to ensuring a superior wound bed for grafting and reliably preventing compartment syndrome. Treatment decisions must account for the prolonged healing times and specific comorbidity burdens in the elderly to optimize outcomes.

## Figures and Tables

**Table 1:** Summarized Comorbidities of the population.

Characteristic/Comorbidity Group	n	%
Age, years (mean ± SD)	74.5 ± 7.6	–
Male sex	30	69.8%
Major Chronic Conditions		
Hypertension	26	60.5%
Dyslipidemia	17	39.5%
Diabetes Mellitus Type 2	9	20.9%
Cardiac Disease *	12	27.9%
Chronic Kidney Disease	5	11.6%
Other Relevant Comorbidities		
Oncologic History	6	14.0%
Chronic Respiratory Disease ^†^	7	16.3%
Neuropsychiatric Disorder ^‡^	8	18.6%
Degenerative Osteoarticular Disease	5	11.6%

* Cardiac Disease is a composite that includes atrial fibrillation, ischemic heart disease, and heart failure. ^†^ Chronic Respiratory Disease combines COPD and Asthma. ^‡^ Neuropsychiatric Disorder combines anxious-depressive disorders, cognitive impairment, and cerebrovascular disease.

**Table 2:** Number of burns with clinical infection.

N	Microorganism
6	*Pseudomonas* spp.
3	* Staphylococcus aureus *
2	*Enterococcus faecium*
2	*Enterobacter cloacae*
1	*Enterococcus faecalis*
1	*Enterococcus avium*
1	*Escherichia coli*
1	*Proteus mirabilis*
1	*Klebsiella pneumoniae*
1	*Klebsiella oxytoca*
1	*Corynebacterium tuberculostearicum*
1	*Morganella morganii*
1	*Mucor* spp.
1	*Candida* spp.

**Table 3:** Causes of death.

N	Causes
3	Multiple Organ Dysfunction
2	Septic Shock
2	Respiratory Insufficiency due to hyperhydration
2	Bronchoaspiration
1	Tension Pneumothorax
1	Massive Intestinal ischemia

## Data Availability

The raw data supporting the conclusions of this article will be made available by the authors on request.

## Abbreviations

The following abbreviations are used in this manuscript:

NXB	Nexobrid
TBSA	Total Body Surface Area
FFP	Fresh Frozen Plasma
RBC	Red Blood cell Concentrate
